# Relaxation techniques as an intervention for chronic pain: A systematic review of randomized controlled trials

**DOI:** 10.1016/j.heliyon.2021.e07837

**Published:** 2021-08-20

**Authors:** Sara Magelssen Vambheim, Tonje Merete Kyllo, Sanne Hegland, Martin Bystad

**Affiliations:** a Department of Psychology, UiT The Arctic University of Norway, Norway; b Department of Pain Management and Research, Emergency Clinic, Oslo University, Norway; c Department of Geropsychiatry, UNN University Hospital of North Norway, Norway

**Keywords:** Chronic pain, Relaxation techniques, Intervention, Randomized controlled studies

## Abstract

Chronic pain increases the risk of sleep disturbances, depression and disability. Even though medical treatments have limited value, the use of prescription-based analgesics have increased over the recent years. It is therefore important to evaluate the effect of non-pharmacological treatments. A systematic search for studies evaluating the effect of relaxation techniques on chronic pain was conducted. Randomized controlled trials were included. Significant effects on pain, or on pain and one or more secondary outcome measure, were found in 21 studies. Four studies found significant effects on secondary outcome measures only. Four studies showed no significant effects on any outcome measure. Thus, most of the studies reported that relaxation techniques reduced pain and/or secondary outcome measures. However, the included studies have evaluated effects across a wide variety of chronic pain conditions and relaxation techniques. Hence, there is a large degree of heterogeneity among the included studies. This complicates the effect evaluation and makes it difficult to draw a clear and unambiguous conclusion. Relaxation techniques are probably most effective when used through regular and continued practice. Future studies should therefore investigate long-term effects of relaxation technique interventions, evaluate the dose-response relationship and examine efficacy differences across pain conditions and interventions.

## 1 Introduction

Chronic pain of moderate to severe intensity is a widespread phenomenon with a prevalence of 19 % in Europe (Breivik et al., 2006). Pain is defined as chronic when it lasts longer than normal healing time, often characterized as more than three months (Treede et al., 2015). Pain is the most important cause of non-lethal health loss, with muscle and skeletal disorders and migraine causing more than one quarter of the health loss (Steingrimsdottir et al., 2018). Chronic pain may limit social functioning, as it increases the risk of sleeping difficulties (Jank et al., 2017), depression (Bair et al., 2003) and work disability (Landmark et al., 2013). According to findings from a population-based health study in Norway, 50% of the work disability cases are associated with chronic pain (Landmark et al., 2013; Nielsen, 2013). A comprehensive treatment of chronic pain involves a combination of different methods and therapies. Pharmacological treatment alone has limited effect and increases the risk of developing dependence (Nielsen, 2013). According to the biopsychosocial model, pain occurs in a complex interaction between biological, psychological and social factors (Finnerup, 2019). Pain may be understood as an interaction of activated pain fibers, pain interpretation and pain behavior (Molton et al., 2009). Thus, pain is both a subjective experience and a physical sensation with large individual differences (Loeser, 1991). The individual differences can be ascribed to an interplay between context, interpretation and understanding of the pain, and the psychological state of the person experiencing pain (Merskey and Bogduk, 1994). Brain imaging studies have shown that afferent activity and activity in the descending pain pathways is influenced by attention and emotional valence, factors not directly related to pain stimuli (Fillingim, 2017).

Relaxation is one example of a non-pharmacological treatment which is increasingly accepted as an intervention for pain reduction and pain coping (Bushnell et al., 2013). A relaxed condition often involves feelings of psychological and bodily wellbeing and calmness (Turk and Winter, 2005, p. 20). The purpose of relaxation techniques is to decrease the activity of the sympathetic nervous system (Benson, 2000, p. 16), through evoking an opposite reaction to the stress response, namely a relaxation response. Practicing relaxation techniques is associated with reduced blood pressure, oxygen uptake, respiratory frequency, heart frequency and muscle tension (Benson, 2000, 2010). Relaxation techniques have several detectable physiological effects, for example lower cortisol levels and inhibition of inflammatory processes (Chang et al., 2011; Bhasin et al., 2013). Relaxation is stress coping and relieves anxiety (Manzoni et al., 2008). For example Bhasin et al. (2013) showed that stress coping after relaxation practice was better the longer the participants practiced.

There are several different types of relaxation techniques, like meditation, breathing techniques, visualization, autogenic training and progressive muscle relaxation (Payne and Donaghy, 2010, s. 20). One possible explanation of why relaxation techniques relieve chronic pain, is that chronic pain is maintained and increased by psychological stress and physical tensions (Chen and Francis, 2010). It has been estimated that three months of regular practice is necessary to obtain pain reduction (Turk and Winter, 2005, s. 49). The effect of relaxation techniques will rely on individual differences and the type of chronic pain (Payne and Donaghy, 2010, s. 20).

Relaxation is considered one of the most available and cost-effective treatments for chronic pain. Also, there are no known side effects (Nazari et al., 2016). Because of the mentioned advantages and lack of knowledge regarding efficiency, this study aims to present an overview of the studies investigating relaxation techniques as an intervention for chronic pain.

## 2 Methods

We sought to investigate unmitigated relaxation techniques. Studies on mindfulness interventions were therefore not included. There are some overlaps between mindfulness and relaxation techniques. However, they have different agendas. Relaxation techniques aims to reduce stress and tension, while the purpose of mindfulness is to observe and accept these feelings (Dunford and Thompson, 2010).

**Figure 1:**
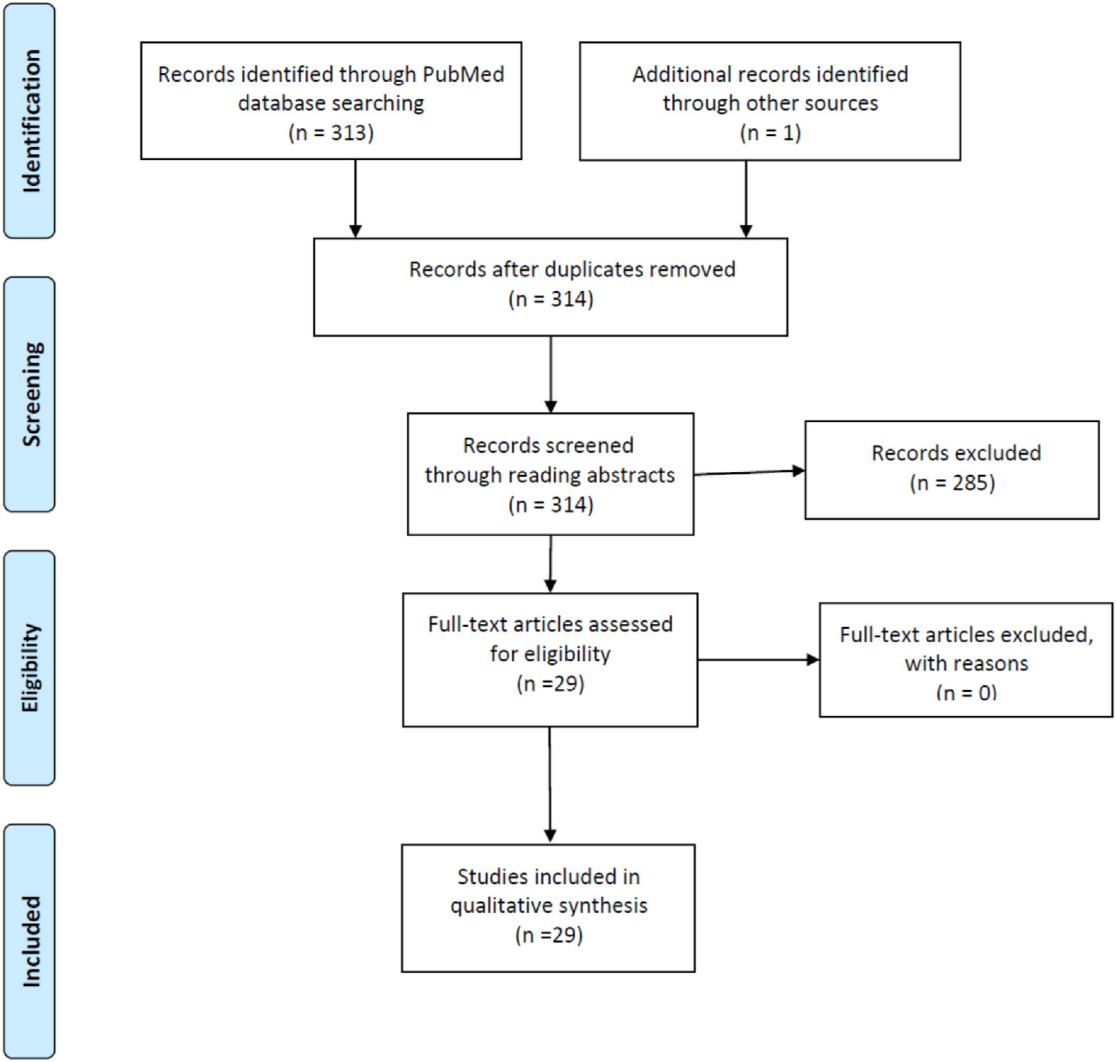
PRISMA flow chart for the selection of studies.

Randomized controlled trials were exclusively included. Furthermore, only studies with relevant outcome measures were included. This involved pain intensity, pain control, pain coping (primary outcome measures) and medical use, physical functioning, quality of life, psychosocial functioning, depression, anxiety, fear- and avoidance behavior (secondary outcome measures). A comprehensive literature search was performed according to the guidelines by Schardt and colleagues (Schardt et al., 2007). Protocol B of Schardt and colleagues was followed in the search process. We had no restrictions regarding gender or country, but we included English articles and studies with participants above 18 years of age. Publications were retrieved from PubMed up to July 15^th^ 2021. The central search strategy included key words to detect studies investigating relaxation techniques as an intervention for chronic pain. The search terms «guided imagery» OR «progressive relaxation» OR «autogenic training» OR «breathing» OR «meditation» were used. The filter “randomized controlled trial” (RCT) was applied. The combination «relaxation» AND «chronic pain» yielded 100 hits, whereof 15 studies were considered relevant. Combining «guided imagery» AND «chronic pain» gave 12 hits, whereof four were considered relevant. «Breathing» AND «chronic pain» yielded 20 hits, whereof three were included. «Meditation» AND «chronic pain» gave 36 hits, whereof three were included. «Autogenic training» AND «chronic pain» gave five hits, whereof three were considered relevant. Records were screened according to the double screening approach (Edwards et al., 2002). Thus, all detected studies were evaluated independently by two of the authors through reading abstracts and discrepancies were resolved through discussion with a third author. One additional study was identified during the process of reviewing the literature. Thus, 29 studies were included in the present review. We only included full-text articles and duplications were excluded. Furthermore, we applied the PRIMSA guidelines (Figure 1). The methods of the analysis and inclusion criteria were specified in advance and documented in a protocol (Moher et al., 2009). The protocol is registered at the Open Science Framework.

## 3 Results

The results are presented in Tables 1, 2, 3, 4, 5. In total 21 studies found significant improvement on pain or pain and one or more other secondary outcome measures. Four studies showed improvement on secondary outcomes, such as anxiety, depression, well-being and coping strategies. Four studies showed no significant improvement on any outcome measures.

**Table 1:** Overview of randomized controlled trials investigating the treatment effect of relaxation technique interventions on chronic pain.

Authors	Sample	Intervention	Control group	Outcome measure	Results	Follow up
Nazari et al. (2016)	MS (N = 75)	Relaxation (n = 25) vs. reflexology (n = 25)	Treatment as usual (n = 25)	Pain intensity.	Significantly lower pain intensity in the relaxation and reflexology group compared to control group.	No significant differences between the three groups two months after the intervention.
Thorsell et al. (2011)	Chronic Pain (N = 90)	Relaxation (n = 35) vs. ACT (n = 35)	Natural history	Quality of life, Pain acceptance, level of activity, depression, anxiety, pain intensity.	Significantly reduced depression and anxiety symptoms.	Significant lower depression and anxiety after 12 months.
Rambod et al. (2014)	Hemodialysis patients (N = 81)	Relaxation (n = 41)	Treatment as usual (n = 40)	Pain intensity, quality of life.	Significantly lower pain intensity and improved quality of life.	No follow-up.
Turner (1982)	Chronic low-back pain (N = 36)	Relaxation in group therapy (n = 14) vs. CBT group therapy (n = 13)	Wait-list control (n = 9)	Pain intensity, physical functioning, depression.	Significant improvement on all outcome measures.	Significant reduction in pain intensity after one month. Increased pain rate after 1,5-2 years
Stuckey et al., (1986)	Chronic low-back pain (N = 24)	Relaxation (n = 8) vs. EMG-biofeedback (n = 8)	Placebo (n = 8)	Pain intensity, daily functioning.	Significant improvement on all outcome measures.	No follow-up.
Shariat et al. (2019)	Chronic low-back pain (N = 72)	Relaxation (19) vs. Exercise (19) vs. Exercise and relaxation (19)	Control group (19)	Pain intensity, range of motion, anxiety, quality of life.	Significant effect on pain intensity and anxiety in all treatment groups. Significant effect on range of motion in Exercise and Exercise and relaxation groups. Significant effect on quality of life in Relaxation and Exercise and Relaxation groups.	Significant effect on pain intensity and anxiety after 12 weeks in all treatment groups. Significant effect on range of motion in Exercise and relaxation group after 12 weeks. Significant effect on quality of life in Relaxation and Exercise and Relaxation groups after 12 weeks.
Gustavsson and von Koch (2006)	Chronic neck pain (N = 29)	Relaxation (n = 13)	Treatment as usual (n = 16)	Pain intensity, physical functioning, depression and anxiety.	Significantly reduced pain intensity.	Significant lower pain intensity after 20 weeks.
Rokicki et al. (1997)	Chronic tension headache (N = 44)	Combined relaxation and EMG-biofeedback-training (n = 30)	Natural history group (n = 14)	Intensity and frequency of headaches.	Significantly reduced frequency of headaches.	No follow-up.
Coppieters et al. (2016)	Chronic WAD (N = 15) and FM (N = 20)	Relaxation	Healthy controls (n = 22)	Pain intensity.	Significantly improved pain-modulation in both groups.	No follow-up.
Chen and Francis (2010)	Musclepain and migraine (N = 19)	Relaxation (n = 11)	Wait-list controls (n = 8)	Pain intensity, quality of life, mental health, sleep.	No significant effects.	No follow-up.
Lauche et al. (2013)	Chronic neckpain (N = 61)	Massage (n = 30) vs. relaxation (n = 31)	Baseline	Pain intensity, quality of life, mental health.	Significantly reduced pain intensity.	No-follow up
Viljanen et al. (2003)	Chronic neck pain (N = 340)	Relaxation (n = 110) vs. dynamic muscle training (n = 111)	Treatment as usual (n = 119)	Pain intensity.	No significant effects.	No significant improvement after 3,6 and 12 months.
Söderberg et al. (2011)	Chronic tension headache (N = 90)	Relaxation (n = 30) vs. acupuncture (n = 30) vs. physical training (n = 30)	Healthy controls (n = 88)	Qualitiy of life, sleep.	Significant improvement in quality of life and sleep.	Significant improvement after 3 and 6 months.
Hasson et al. (2004)	Long lasting muscle pain (N = 120)	Relaxation (n = 55) vs. massage (n = 62)	Baseline	Muscle pain, self-reported health, mental energy.	No significant effects.	No significant improvement after three months.
Ter Kuile et al. (1996)	Chronic headache (N = 144)	Self-hypnosis (n = 72) vs. relaxation (n = 72)	Baseline	Pain intensity.	Significantly reduced pain intensity.	Significant improvement after 6 months.

Note: Biofeedback is a mind-body technique in which patients with chronic pain can modify some of the body functions by receiving information from electrical sensors. EMG (Electromyography) is a biofeedback method that measures and gives feedback about muscle tension. Self-hypnosis is related to relaxation, but relies on changing pain perception rather than eliciting a relaxation response. CBT (cognitive behavioral therapy) aims to change patterns of behavior and thinking. Dynamic muscle training is a type of strength training with light weights (1–3 kg). ACT (Acceptance and commitment therapy) aims to accept thoughts and feelings in the present moment and change behavior in accordance with values. Reflexology is a type of massage with application of specific pressure points on the feet and hands.

**Table 2:** Overview of randomized controlled trials investigating the treatment effect from interventions using «guided imagery» on chronic pain.

Authors	Sample	Intervention	Control	Outcome measure	Results	Follow up
Verkaik et al. (2014)	FM (N = 53)	Guided imagery (n = 26)	Supportive group therapy (n = 27)	Pain intensity, self-efficacy, physical status.	No significant effects.	No significant improvement after 6 weeks.
Lewandowski (2004)	Patients with chronic pain (N = 42)	Guided imagery (n = 21)	Natural history (n = 21)	Pain intensity.	Significantly reduced pain intensity.	No follow-up.
Baird and Sands (2004)	Females (>65 years) with osteoarthritis (N = 28)	Guided imagery and progressive relaxation (n = 18)	Natural history (n = 10)	Pain intensity, mobility.	Significantly reduced pain intensity and improved mobility.	No follow-up.
Torres et al. (2018)	Females with FM (N = 56)	Relaxation and guided imagery (n = 33)	Natural history (n = 23)	Well-being, functional status, anxiety/depression, pain-perception.	Significantly improved well-being and reduced anxiety.	No significant improvement after 3 months.
Onieva-Zafra et al. (2015)	FM (N = 60)	Relaxation (n = 30)	Natural history (n = 30)	Pain intensity and depression.	Significantly reduced pain intensity and depression.	No significant improvement after 8 weeks.

**Table 3:** Overview of randomized controlled trials investigating the treatment effect of interventions using the relaxation technique «breathing» on chronic pain.

Authors	Sample	Intervention	Control	Outcome measure	Results	Follow up
Kapitza et al. (2010)	Chronic low-back pain (N = 42)	Respiratory bio-feedback (n = 21)	Placebo (n = 21)	Pain intensity, functional status, relaxation index.	Significantly reduced pain intensity.	Significant improvement after three months.
Mehling et al. (2005)	Patients with chronic low-back pain (N = 36)	Breathing (n = 16) vs. physical therapy (n = 12)	Baseline	Pain intensity, physical function.	Significantly reduced pain intensity.	Significant lower pain intensity and physical function after 6 months.
Tekur et al. (2008)	Females with chronic low-back pain (N = 80)	Intensive short-term yoga program (n = 40)	Physical training (n = 40)	Pain intensity.	Significantly reduced pain intensity.	No follow-up.

**Table 4:** Overview of randomized controlled trials investigating the treatment effect of interventions using the relaxation technique «meditation» on chronic pain.

Authors	Sample	Intervention	Control	Outcome measure	Results	Follow up
Kiran et al. (2014)	Chronic tension headache (N = 50)	Meditation (n = 30)	Treatment as usual (n = 20)	Pain intensity, pain frequency, medications.	Significant improvement on all outcome measures.	No follow-up.
Jeitler et al. (2015)	Chronic neck pain (N = 89)	Meditation (n = 45) vs. physical exercise (n = 44)	Baseline	Pain intensity, stress, well-being, depression/anxiety, quality of life.	Significantly reduced pain intensity.	No follow-up.
Michalsen et al. (2016)	Chronic low-back pain (N = 68)	Meditation (n = 32) vs. physical exercise (n = 36)	Baseline	Pain intensity, stress, well-being, quality of life.	Significantly reduced pain intensity and stress.	No follow-up.

**Table 5:** Overview of randomized controlled trials investigating the treatment effect of interventions using the relaxation technique «autogenic training» on chronic pain.

Authors	Sample	Intervention	Control	Outcome measure	Results	Follow up
Jensen et al. (2010)	MS and chronic pain (N = 15)	Autogenic training and cognitive restructuring (n = 15)	Education (n = 15)	Pain intensity, catastrophic thinking, pain-distraction, worst pain.	Significantly reduced pain intensity, worst pain and pain distraction.	No follow-up.
Jensen et al. (2010)	Spinal cord injury (N = 37)	EMG biofeedback relaxation (n = 14) vs. autogenic training (n = 23)	Baseline	Pain intensity, depressive symptoms, perceived pain-control.	Significantly reduced pain intensity and improved perceived pain-control in the autogenic training group.	Significant lower pain intensity in the autogenic training group after three months.
Ter Kuile et al. (1994)	Recurrent headaches (N = 146)	Autogen trening (n = 44) vs. Self-hypnosis (n = 46)	Wait-list controls (n = 56)	Pain intensity, medications, Pain intensity, stress.	Significantly reduced pain intensity in both intervention groups.	Significant lower pain intensity in both intervention groups after six months.

## 4 Discussion

The majority of the included studies showed significant pain reduction, or significant pain reduction and improvement on one or more secondary outcome measure. In total four of the included studies showed no significant effect on any outcome measure.

There are several potential explanations to why relaxation techniques reduce chronic pain. One possibility is that relaxation techniques reduce chronic pain through triggering pain inhibitory processes in the brain, which further influence the pain experience (Melzack and Wall, 1996, s. 339). According to the neuromatrix theory (Melzack, 2001) pain signals to the brain are inhibited by relaxation because the amount of pain signals to the brain are reduced. Thus, reduced stress after practicing relaxation techniques may influence the experience of pain (Edwards et al., 2016). Relaxation techniques may thus contribute to stress coping, with a following reduced pain experience (Sættem and Stiles, 2008).

It is likely that the type of chronic pain is important for the pain relieving effect of relaxation techniques. This was shown in the study by Coppieters et al. (2016), where patients with whiplash-associated disorder (WAD) showed larger pain reduction from relaxation techniques compared to patients with fibromyalgia (FM). This illustrates the importance of comparing the efficacy of relaxation techniques across pain conditions and disorders.

Whether relaxation techniques are more advantageous than other non-pharmacological treatments remains unclear, even though most of the included studies suggests that relaxation techniques add value to the treatment and management of pain. Shariat et al. (2019) investigated the efficacy on pain intensity, anxiety, quality of life (QOL) and range of motion (ROM) in four different groups (Relaxation, Exercise, Exercise combined with relaxation, Control). The patients had chronic low back pain and were randomized to one of the four groups. Significant effects were found on pain intensity and anxiety in all treatment groups after six and twelve weeks. ROM was significantly improved in the Exercise group and the Exercise combined with relaxation group. However, at a 12-week follow-up, the effect on ROM was only still present in the Exercise combined with relaxation group. QOL was significantly enhanced in the Relaxation group and the Exercise combined with relaxation group at six and twelve weeks. Accordingly, combining exercise and relaxation training may be preferable over exercise or relaxation training only.

Several of the included studies reported that the effect of the relaxation intervention declined after the end of the intervention (Turner, 1982; Onieva-Zafra et al., 2015; Torres et al., 2018). This implies that relaxation techniques have short-lived effects on chronic pain and needs to be practiced continually if the effect should be maintained. However, 13 of the studies examined in this review did not include a follow-up measure. This complicates the evaluation of the duration of treatment effects beyond the intervention period. Furthermore, there is no consensus on which type of relaxation technique works best. Although the present review cannot draw a conclusion on the dose of relaxation training needed to achieve significant pain relief in chronic pain patients, previous research has suggested 90 days of regular practice (Turk and Winter, 2005, p. 49) and a general recommendation is to practice daily for at least 10 min (Benson, 2010 p. 167). More evidence from RCTs designed to compare treatment efficacy of different doses and types of relaxation training, as well as the duration of the treatment effect, is required for future studies to address these issues.

The most frequently employed techniques among the included studies were progressive relaxation (11 studies), autogenic training (7 studies) guided imagery (6 studies). Breathing exercises are easy to learn and apply and may be more time efficient than other relaxation techniques (e.g 5 min of practice may be sufficient). This has been demonstrated in an experimental study by Busch et al. (2012). However, there is a need for more RCTs to investigate the efficacy of breathing exercises and chronic pain more thoroughly.

The included studies employed a wide array of methods and designs and included several different patient groups. Furthermore, intra-individual sensitivity to relaxation techniques may be an obstacle in pursuing its effect on pain. These issues complicates explaining efficacy differences reported in different studies. Although one specific relaxation technique cannot be recommended based on the current evidence, our findings indicate that relaxation techniques may be an adjuvant intervention for chronic pain patients.

The results from some of the studies that employed the same relaxation technique on the same patient group are inconsistent. For example, Viljanen et al. (2003), Gustavsson and von Koch (2006) and Lauche et al. (2013) investigated if patients with chronic neck pain would benefit from progressive muscle relaxation training. Treatment efficacy were insignificant in Viljanen et al. (2003) study, but significant in Gustavsson and von Koch (2006) and Lauche et al. (2013). One potential explanation is that the intervention programs used in the three studies had some differences, with diverse amounts of training and numbers of visits throughout the intervention. The effect reported by Lauche and colleagues was only marginally significant, and the effect was not significantly different from cupping massage, which was given to the other treatment group. These inconsistencies and findings of small effects calls for more documentation through robust large sample studies. However, according to previous reviews (e.g Kwekkeboom and Gretarsdottir, 2006) the most frequently supported technique for chronic low-back pain and arthritis pain is progressive muscle relaxation.

### 4.1 Methodological limitations

This review has some methodological limitations that needs to be addressed. One limitation is that only RCTs were included. RCTs are considered the gold standard for intervention research (Concato et al., 2000) due to the strict requirements of inclusion criteria and their standardized, experimental design. However, RCT studies are less capable of handling individual needs and adjustments and relaxation techniques will often give the best results if they are individually tailored (Payne and Donaghy, 2010, s. 20). This challenges the generalizability of findings from RCTs. Future studies should therefore include more than just RCT-studies. Another limitation is that we did not rely on a standardized quality assessment of the RCT studies. However, we followed the PRISMA review protocol.

The included studies investigated different pain conditions and different relaxation techniques. Thus, the degree of heterogeneity is large. This complicates the effect evaluation across the studies and makes it difficult to draw an unambiguous conclusion. Additionally, some of the included studies have a relatively short duration (1–8 weeks) (Chen and Francis, 2010; Rambod et al., 2014; Verkaik et al., 2014; Tekur et al., 2008). Relaxation techniques require continual practice over time, and the effect may improve with time (Turk and Winter, 2005, s. 59, Benson et al., 2010, s. 205). For instance, a study by Perlman et al. (2010) found that expert meditators (with over 10 000 h of meditation) displayed higher tolerance for experimental pain, compared to novice meditators. Thus, there may be a “dose-response” relationship between practice of relaxation and effects on chronic pain. The length of interventions on chronic pain should therefore be carefully evaluated, to ensure that the real effect can be initiated and registered. Moreover, the reliability of the findings may be undermined by small sample sizes in several of the included studies.

## 5 Conclusion

Several of the included studies show that relaxation techniques reduce pain and influence secondary outcome measures in patients with chronic pain. This implies that relaxation techniques may be a valuable addition to programs designed for chronic pain management. However, relaxation techniques should not be used as a stand-alone treatment, but rather supplement established treatment programs. The effect tends to decrease over time and continuation of practice may be necessary for pain reduction maintenance. This is not surprising, given that the relaxation-response is the underlying mechanism of pain reduction achieved through these techniques. The relaxation response has to be regularly activated to exert effects on the nervous system and hence on pain experience. Relaxation techniques are probably most effective in the long run, when used regularly over time. Future studies should therefore investigate the long-term effect of longitudinal interventions and evaluate dose-response and differences in efficacy between chronic pain conditions and relaxation interventions.

## Declarations

### Author contribution statement

All authors listed have significantly contributed to the development and the writing of this article.

### Funding statement

This work was supported by 10.13039/100017619UiT The arctic university of Norway.

### Data availability statement

Data included in article/supplementary material/referenced in article.

### Declaration of interests statement

The authors declare no conflict of interest.

### Additional information

No additional information is available for this paper.
